# Pressure gradients calculated from PC-MRI, SPIV and CFD velocity data in a phantom model: comparison with catheter-based pressure measurement

**DOI:** 10.1186/1532-429X-14-S1-W34

**Published:** 2012-02-01

**Authors:** Iman Khodarahmi, Mostafa Shakeri, Melanie Kotys-Traughber, Stefan Fischer, Keith M Sharp, Amir Amini

**Affiliations:** 1 Electrical Engineering, University of Louisville, Louisville, KY, USA; 2 Mechanical Engineering, Univeristy of Louisville, Louisville, KY, USA; 3 MR Research, Philips Healthcare, Cleveland, OH, USA

## Background

Peripheral arterial disease (PAD) is a common manifestation of atherosclerosis and is defined as any pathologic process causing obstruction to blood flow in the arteries outside the heart; mainly the arteries supplying the lower extremities. Phase-contrast MRI (PC-MRI) provides a powerful and non-invasive method to acquire spatially registered blood velocity. The velocity field, then, can be used to derive other clinically useful hemodynamic parameters, such as blood pressure gradients.

## Methods

**Figure 1:**
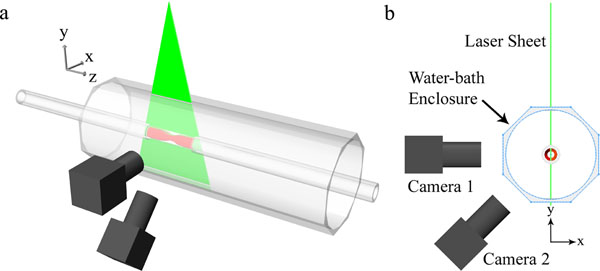
Schematic side view (a) and isometric view (b) of the SPIV apparatus.

Herein, pressure gradient across an axisymmetric Gaussian-shaped 87% area stenosis phantom was estimated by solving the pressure-Poisson equation (PPE). The velocity field needed to solve the pressure equation was obtained using PC-MRI and Stereoscopic Particle Image Velocimetry (SPIV). Steady flow rate of 46.9 ml/s, corresponding to an inlet Reynolds number of 160, was used which mimics the Reynolds number of human common iliac artery. Sagittal PC-MRI images were acquired using a standard 2D phase contrast sequence with Cartesian read-out, through-plane velocity encoding, and velocity compensation in all three directions on a 3T TX Achieva Philips MRI scanner with slice thickness = 2 mm, resolution = 1 × 1 mm, TE/TR = 3.0/4.0 ms, field of view = 192 × 64 mm, and velocity encoding (Venc) = 120 cm/s. For SPIV purposes, a 532-nm laser light sheet was passed parallel to the axis of the phantom to illuminate the flowing fluorescent particles (Fig. 1). A set of image pairs were captured using two cameras looking at the phantom at different angles and the fluid velocity was extracted using a cross-correlation scheme, yielding a nominal spatial resolution of 0.168 mm for the velocity data.

Computational Fluid Dynamics (CFD) simulation of the same flow was performed on the same geometry using the CFD software package Fluent 12.1 based on a finite volume scheme.

## Results

**Figure 2:**
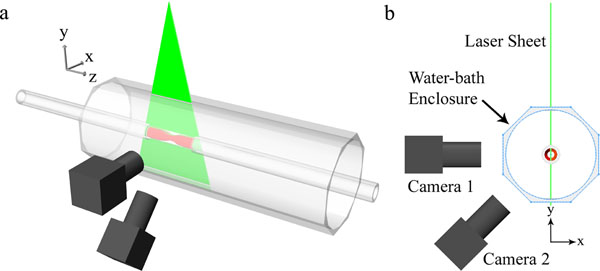
Pressure profiles along the centerline of the phantom calculated from different methods.

The results of the PPE solution using the PC-MRI and SPIV velocity data are shown in Fig 2.

The Poisson equation was also solved using the CFD velocities, regridded to a rectangular mesh of the same grid resolution as PC-MRI. The pressure distribution obtained directly from the Fluent software is also shown for comparison. As shown in figure 2, good agreement exits between pressures calculated from different methods.

## Conclusions

Pressure gradients calculated from PC-MRI data is comparable with those obtained from other experimental and numerical methods. Direct pressure measurement using two simultaneous catheters placed proximal and distal to the stenosis is currently under investigation and will be added as the ground truth to the discussed methods.

## Funding

This work was supported in part by the National Science Foundation under Grant 0730467 and by an innovative grant from the Clinical and Translational Research Program of the University of Louisville.

